# Antibiotic prophylaxis and its effect on postprocedural adverse events in endoscopic retrograde cholangiopancreatography for primary sclerosing cholangitis

**DOI:** 10.1002/jgh3.12846

**Published:** 2022-12-05

**Authors:** Arvid Gustafsson, Lars Enochsson, Bobby Tingstedt, Greger Olsson

**Affiliations:** ^1^ Department of Research and Development and Department of Surgery Central Hospital Växjö Sweden; ^2^ Department of Clinical Sciences Lund, Surgery, Lund University and Department of Surgery Skåne University Hospital Lund Sweden; ^3^ Department of Surgical and Perioperative Sciences, Surgery Umeå University Umeå Sweden

**Keywords:** adverse events, antibiotic prophylaxis, endoscopic retrograde cholangiopancreatography, primary sclerosing cholangitis

## Abstract

**Background and Aim:**

Primary sclerosing cholangitis (PSC) is characterized by multiple strictures of the biliary tree. Patients with PSC frequently require repeated endoscopic retrograde cholangiopancreatography (ERCP) procedures. These procedures are encumbered by an increased incidence of infectious adverse events such as cholangitis. Evidence regarding whether antibiotic prophylaxis (AP) should be administered is sparse; however, prophylaxis is recommended. We aimed to determine whether AP affects the rate of postprocedural infectious and overall adverse events.

**Methods:**

We conducted a retrospective cohort study and extracted all ERCP procedures with indicated PSC performed between 1 January 2006 and 31 December 2019, which were registered in the Swedish Registry for Gallstone Surgery and ERCP (GallRiks). The exclusion criteria were incomplete 30‐day follow‐up, non‐index procedures, or ongoing antibiotics. The main outcomes were postprocedural infectious adverse events and overall adverse events at the 30‐day follow‐up.

**Results:**

A total of 2144 procedures with indication of PSC were eligible for inclusion. AP was administered in 1407 (66%) of these procedures. Patients receiving AP were slightly younger (44 *vs* 46 years, *P* = 0.005) and had more comorbidities (ASA ≥3, 19.8% *vs* 13.6%; *P*  < 0.001). Procedures with AP demonstrated an infectious adverse event rate of 3.3% compared to 4.5% for non‐AP procedures (*P* = 0.19). Postprocedural infectious adverse events (odds ratio [OR] 0.76, 95% confidence interval [CI] 0.48–1.21) and overall adverse events (OR 0.79, 95% CI 0.60–1.04) did not differ between AP and non‐AP.

**Conclusion:**

Patients with PSC who undergo ERCP have the same frequency of adverse events regardless of whether AP was used.

## Graphical abstract

Patients with primary sclerosing cholangitis are encumbered with increased incidence of infectious adverse events when undergoing endoscopic retrograde cholangiopancreatography procedures. In this retrospective cohort study, we investigated the use of antibiotic prophylaxis in 2144 such procedures. We found no reduction in adverse events even if prophylaxis was administered.

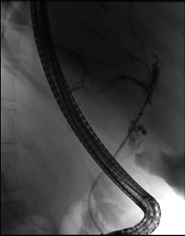

## Introduction

Primary sclerosing cholangitis (PSC) is a chronic cholestatic liver disease with a prevalence of up to 30 cases per 100 000 individuals. The disease is frequently associated with concurrent inflammatory bowel disease, with ulcerative colitis being the most common manifestation.^1^ PSC is characterized by multiple biliary strictures, with a risk of developing liver cirrhosis and cholangiocarcinoma. Endoscopic retrograde cholangiopancreatography (ERCP) is the cornerstone in the diagnosis and treatment of PSC.^2^, ^3^, ^4^, ^5^ For patients with PSC, ERCP procedures are burdened with higher rates of adverse events than ERCP procedures for other indications.^2^, ^6^, ^7^, ^8^, ^9^, ^10^, ^11^, ^12^ PSC, in particular, is shown to be an independent risk factor for post‐ERCP cholangitis (PEC).^10^, ^13^ The adverse events in ERCP for PSC are linked to longer duration^12^ and have been shown to be even higher in the event of acute or symptomatic indication^6^, ^7^ or more frequent technical maneuvers such as dilatation of the bile ducts.^6^, ^14^ The underlying cause of the higher rate of adverse events is considered to be the nature of the disease with multiple strictures in the bile ducts, making the possibility of complete biliary drainage more difficult.^9^, ^15^

The higher rates of infectious adverse events observed when ERCP is performed for PSC could theoretically be avoided by administering antibiotic prophylaxis (AP). This is also recommended in several guidelines, including the European Society of Gastrointestinal Endoscopy (ESGE) guidelines.^3^, ^15^, ^16^, ^17^ The basis for recommending AP is that ERCP for PSC represents an occasion where adequate drainage of the biliary tract is rarely achieved. This is a situation in which AP should be considered^15^ based on previous studies of risk factors for infectious adverse events in ERCP.^18^, ^19^ However, evidence to support the recommendation to generally administer AP to patients with PSC undergoing ERCP is scarce.^3^, ^15^

This study aimed to identify the effects of AP on postprocedural adverse events during ERCP for PSC. The primary objective was to evaluate the effects of prophylactic antibiotics on infectious adverse events such as PEC and abscess formation. The secondary objective was to determine whether prophylaxis was associated with overall adverse events.

## Methods

### Study design and population

All ERCP procedures performed in Sweden between 1 January 2006 and 31 December 2019, which were registered in the Swedish Registry for Gallstone Surgery and ERCP (GallRiks), were assessed for inclusion in this nationwide, population‐based cohort study. The included procedures originated from 44 separate centers and clinics. The inclusion criteria were ERCP procedures with an indication of PSC. The same patient could be included more than once because of the possibility of undergoing several ERCP procedures. The exclusion criteria were incomplete 30‐day follow‐up, non‐index procedure, and ongoing antibiotics. A corresponding recorded 30‐day follow‐up was used to compare the rates of infectious and overall postprocedural adverse events with respect to whether prophylactic antibiotics were administered during the ERCP procedure. Other specific adverse events, including PEC, infection with abscesses, pancreatitis, perforation, and bleeding, were also investigated.

**Figure 1:**
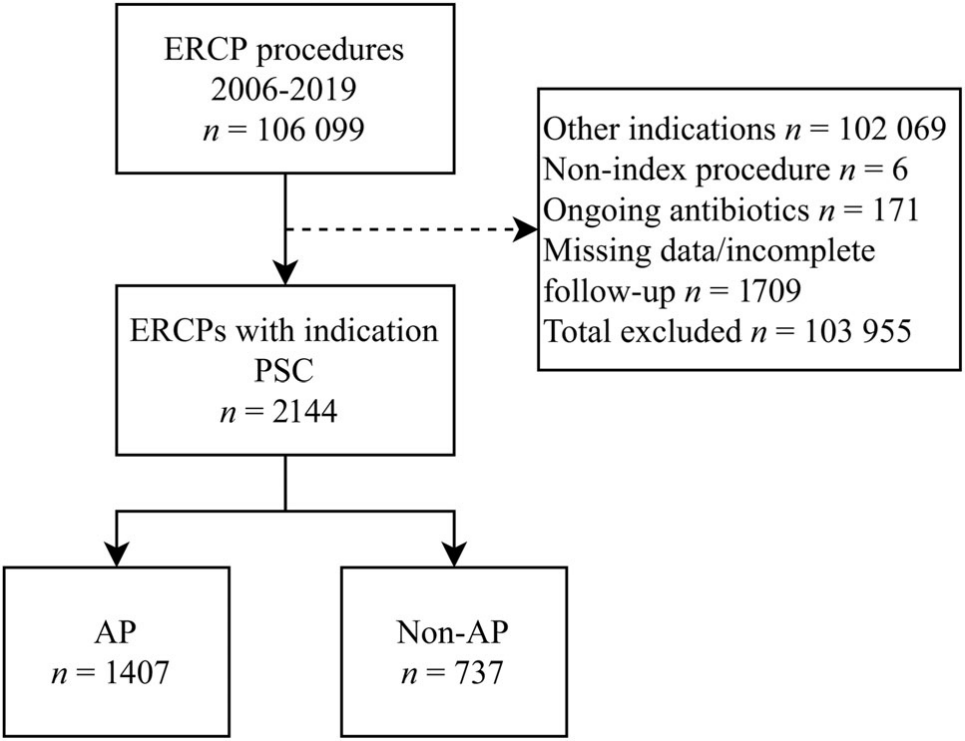
Inclusion/exclusion of procedures and nesting of cohorts. AP, antibiotic prophylaxis; ERCP, endoscopic retrograde cholangiopancreatography; PSC, primary sclerosing cholangitis.

Between 2006 and 2019, 106 099 ERCP procedures were entered into GallRiks. Investigations with missing data (*n* = 1709), non‐index procedures (*n* = 6), ongoing antibiotic therapy (*n* = 171), or indications for ERCP other than PSC (*n* = 103 955) were excluded. After exclusion, 2144 ERCP procedures with indication PSC were eligible for the study. Prophylactic antibiotics were administered in 1407 (66%) of these procedures (Fig. 1).

### The GallRiks registry

The GallRiks registry was founded in 2005 by the Swedish Surgical Society and is supported by the Swedish National Health Authority. It is an online managed registry (www.ucr.uu.se/gallriks) in which data on cholecystectomies and ERCPs are recorded at the time of the procedure. Once recorded, the questionnaire was closed without the possibility for the endoscopist to alter it. In each ERCP procedure, a considerable number of parameters are present for registration, and several parameters are compulsory. Thirty days after ERCP, follow‐up was performed through a review of medical records or a telephone interview. This was typically performed by an appointed non‐physician coordinator of GallRiks at each participating hospital.

The national coverage of ERCP procedures in GallRiks has constantly increased during the study period. The registry reached 99% of Swedish hospitals in 2011 and has since expanded to include more than 90% of procedures performed in Sweden.^20^ Currently, GallRiks contains more than 125 000 ERCP investigations. The registry also retains a high validity,^21^ maintained through regular visits to participating hospitals from independent observers who compare the data in GallRiks with those from medical records.

### Variables

To minimize the risk of confounding among the associations of the numerous parameters in the registry, we selected variables previously shown to be clinically significant for the risk of adverse events in ERCP. We present the selected variables below with their definitions according to GallRiks.

*Age* was dichotomized as over or under 45 years, which represented the mean age of the study population.

*Cannulation failure* is a risk factor for postprocedural adverse events and was defined as the inability to reach deep contact within the bile duct with either a contrast agent or guidewire. However, the reason for this failure cannot be registered in GallRiks. Shallow cannulation, cannulation outside the bile duct, and non‐initiated cannulation process were treated as failed cannulation.

*Pancreatic cannulation* is another known risk factor for postprocedural adverse events. It was defined as unintentional contact of the pancreatic duct with a guidewire or contrast medium.

*Precut sphincterotomy* was defined as an opening technique of the bile duct performed without any guidewire placed in the duct. Both precut sphincterotomy alone or precut followed by regular sphincterotomy were defined as precut.

*Procedure time* was dichotomized as more or less than 55 min, representing the median duration of ERCP procedures.

*Postprocedural adverse events* are registered in GallRiks at follow‐up 30 days after the procedure. These include post‐ERCP pancreatitis (PEP), PEC, abscess formation, and any other adverse event requiring antibiotic treatment, re‐intervention, blood transfusion, or any other condition that causes a prolonged hospital stay or recovery.

*Postprocedural infectious adverse events* include both PEC and abscess formation.

*Antibiotic administration* is registered in GallRiks as prophylaxis or therapy. Additionally, it is noted whether antibiotic treatment was administered until the 30‐day follow‐up. The type or dose of antibiotics is not registered.

### Statistical analysis

Categorical variables are presented as absolute numbers with frequencies (percentages), and Pearson's chi‐square test was used to calculate differences. Quantitative values are expressed as mean ± SD, and Student's *t*‐test was used for the calculation. A *P*‐value less than 0.05 in a two‐sided test was considered statistically significant.

We used multivariable logistic regression analysis to test the association between prophylactic antibiotic use and 30‐day postprocedural infectious an overall adverse events. These results are expressed as odds ratios (ORs) with the corresponding 95% confidence intervals (CIs). The variables were first tested for univariability to detect those relevant for inclusion in the multivariable analysis according to Hosmer's purposeful selection model.^22^ Backward stepwise regression was then used to eliminate variables with an *α* >0.1 until the model was fit. Attention was paid to potential variables with interactions in the model to prevent them from being excluded. JMP version 15.2.0 (64‐bit, SAS Institute, Cary, NC, USA) was used for the analysis.

### Ethical considerations

This study was approved by the Swedish Ethical Review Authority (DNR: 2019‐03717).

## Results

Of the 44 centers, a single university center accounted for 37.2% of the 2144 ERCP procedures. Five high‐volume university centers accounted for 63.1% of the procedures. There were no statistical differences between high‐ and low‐volume centers regarding overall adverse events (14.6% *vs* 14.3%, *P* = 0.86) or postprocedural infectious complications (3.7% *vs* 3.8%, *P* = 0.91). The demographic data of the patients for included procedures are presented in Table 1. There were no differences in the distribution of females and males. Patients receiving prophylactic antibiotics were slightly younger and had a higher proportion of comorbidities according to the ASA classification (Table 1).

**Table 1:** Baseline characteristics

	AP *n* = 1,407 (66%)	Non‐AP *n* = 737 (34%)
Sex, *n* (%)		
Female	461 (32.8)	243 (33.0)
Male	946 (67.2)	494 (67.0)
Age, mean (SD), years	44.1 (15.0)	46.0 (16.0)
ASA, *n* (%)		
1–2	1,128 (80.2)	637 (86.4)
3–4	279 (19.8)	100 (13.6)
Setting, *n* (%)		
Acute	77 (5.5)	56 (7.6)
Elective	1,330 (94.5)	681 (92.4)

AP, antibiotic prophylaxis; ASA, American Society of Anesthesiologists classification.

In ERCP procedures for patients with PSC in whom AP was administered, postprocedural infectious adverse events occurred with a frequency of 3.3% compared with 4.5% if AP was not administered. Postprocedural adverse events occurred in 15.6% of procedures without AP compared with 13.9% procedures with AP (Table 2).

**Table 2:** Complications at 30‐day follow‐up

	AP *n* = 1,407 (66%)	Non‐AP *n* = 737 (34%)	*P*‐value
In general, *n* (%)			
Infectious complications	47 (3.3)	33 (4.5)	0.19
Overall adverse events	195 (13.9)	115 (15.6)	0.28
Specific, *n* (%)			
Cholangitis (PEC)	45 (3.2)	29 (3.9)	0.37
Infection with abscess	2 (0.1)	4 (0.5)	0.10
Pancreatitis	109 (7.8)	54 (7.3)	0.73
Perforation	14 (1.0)	6 (0.8)	0.68
Bleeding	9 (0.6)	3 (0.4)	0.49
Antibiotic treatment	97 (6.9)	69 (9.4)	**0.04**
Re‐ERCP	26 (1.9)	16 (2.2)	0.61

*Note*: Bold values are statistically significant.

AP, antibiotic prophylaxis; PEC, post‐ERCP cholangitis.

No differences were seen with respect to other adverse events such as pancreatitis, perforation, or bleeding, regardless of whether AP was administered. However, unadjusted, significantly more patients were treated with antibiotics during the 30‐day follow‐up if no AP was administered at the time of ERCP (Table 2). No differences in reintervention (re‐ERCP) were observed between the groups (Table 2).

Regarding the risk factors for infectious adverse events, only higher age was significant in the multivariable model (Table 3). The significant risk factors for postprocedural adverse events identified in the association analysis were pancreatic cannulation and a longer procedure duration (Table 4). Gender and age were not found to be risk factors for overall adverse events.

**Table 3:** Associations between patient‐, indication‐, and procedure‐related risk variables and postprocedural infectious complications

	Number of cases (%)/controls	Univariable OR (95% CI)	Multivariable OR (95% CI)
Sex			
Female	20 (2.8)/684	1.00 (Ref.)	1.00 (Ref.)
Male	60 (4.2)/1,380	1.49 (0.89–2.49)	1.61 (0.96–2.70)
Age, years			
>45	43 (4.6)/893	1.52 (0.97–2.38)	**1.58 (1.00–2.48)**
≤45	37 (3.1)/1,167	1.00 (Ref.)	1.00 (Ref.)
ASA			
1–2	61 (3.5)/1,704	1.00 (Ref)	—
3–4	19 (5.0)/360	1.47 (0.87–2.50)	
Cannulation of bile duct			
Succeeded	75 (3.8)/1,920	1.13 (0.45–2.83)	
Failed	5 (3.4)/144	1.00 (Ref.)	—
Cannulation of pancreatic duct			
Yes	22 (5.1)/407	1.54 (0.93–2.55)	1.60 (0.97–2.66)
No	58 (3.4)/1,657	1.00 (Ref.)	1.00 (Ref.)
Precut sphincterotomy			
Yes	5 (5.9)/80	1.65 (0.65–4.20)	—
No	75 (3.6)/1,984	1.00 (Ref)	
Procedure time, min			
>55	42 (4.0)/1000	1.18 (0.75–1.84)	—
≤55	38 (3.5)/1064	1.00 (Ref)	

*Note*: Bold values are statistically significant.

ASA, American Society of Anesthesiologists classification; CI, confidence interval; OR, odds ratio.

**Table 4:** Associations between patient‐, indication‐, and procedure‐related risk variables and overall adverse events

	Number of cases (%)/controls	Univariable OR (95% CI)	Multivariable OR (95% CI)
Sex			
Female	96 (13.6)/608	1.00 (Ref.)	—
Male	214 (14.9)/1,226	1.11 (0.85–1.43)	
Age, years			
>45	131 (14.0)/805	1.00 (Ref.)	—
≤45	179 (14.9)/1,025	1.07 (0.84–1.37)	
ASA			
1–2	249 (14.1)/1,516	1.00 (Ref)	—
3–4	61 (16.1)/318	1.17 (0.86–1.58)	
Cannulation of bile duct			
Succeeded	287 (14.4)/1,708	1.00 (Ref)	
Failed	23 (15.4)/126	1.09 (0.68–1.72)	—
Cannulation of pancreatic duct			
Yes	102 (23.8)/327	**2.26 (1.73–2.95)**	**2.21 (1.69–2.88)**
No	208 (12.1)/1,507	1.00 (Ref.)	1.00 (Ref.)
Precut sphincterotomy			
Yes	18 (21.2)/67	1.63 (0.95–2.78)	—
No	292 (14.2)/1,767	1.00 (Ref)	
Procedure time, min			
>55	170 (16.3)/872	**1.34 (1.05–1.71)**	**1.42 (1.09–1.85)**
≤55	140 (12.7)/962	1.00 (Ref)	1.00 (Ref)

*Note*: Bold values are statistically significant.

Abbreviations: OR, odds ratio; CI, confidence interval; ASA, American Society of Anesthesiologists classification.

When adjusted for potential confounders, we found a nonsignificant OR of 0.76 for infectious adverse event rates when AP was administered. A similar finding was reached for overall adverse event rates, where the nonsignificant OR was 0.79 when prophylactic antibiotics were administered (Table 5).

**Table 5:** Multivariable analysis of antibiotic prophylaxis and primary outcomes

Postprocedural infectious complications			
	Number of cases (%)/controls	Univariable OR (95% CI)	Multivariable OR (95% CI)
AP	47 (3.3)/1,360	0.74 (0.47–1.16)	0.76 (0.48–1.21)
Non‐AP	33 (4.5)/704	1.00 (Ref.)	1.00 (Ref)

Postprocedural adverse events			
	Number of cases (%)/controls	Univariable OR (95% CI)	Multivariable OR (95% CI)
AP	195 (13.9)/1,212	0.87 (0.68–1.12)	0.79 (0.60–1.04)
Non‐AP	115 (15.6)/622	1.00 (Ref.)	1.00 (Ref.)

AP, antibiotic prophylaxis; CI, confidence interval; OR, odds ratio.

## Discussion

We were unable to identify any evidence that the administration of prophylactic antibiotics to patients who underwent ERCP for the diagnosis of PSC would reduce overall or infectious adverse events. There were no advantages in specific infectious adverse events such as PEC or abscess formation, nor could any influence be demonstrated on general adverse events after ERCP using prophylactic antibiotics. These findings might indicate that the recommendations for using prophylactic antibiotics in ERCP procedures performed with the indication of PSC could be questioned. However, patients with PSC still have an increased risk of PEC despite receiving AP.

The adverse event rates in our study for both groups (with or without AP) were higher than those in studies on adverse events of ERCP procedures in general.^11^, ^19^ Compared to a systematic review by Andriulli *et al*. studying regular ERCP procedures, both the overall adverse event rate and the PEC rate were doubled in patients with PSC.^11^ This is in line with previous studies reporting mainly similar adverse event rates; but, especially the risk of PEC is consistently elevated.^2^, ^7^, ^8^, ^9^, ^10^, ^12^, ^14^ These previous findings, in line with ours, suggest that AP is somewhat ineffective in preventing PEC; however, this does not necessarily indicate that patients with PSC do not need AP. Hypothetically, AP should be administered more selectively in patients with PSC with the greatest risk of PEC. Alternatively, PSC patients might need an extended duration of AP or another type of antibiotic.

The higher risk of PEC has been discussed and mentioned in the ESGE guidelines as being caused by the expected more frequent situation with incomplete biliary drainage in patients with PSC.^3^, ^15^ This assumption is possibly based on the known risk factors for PEC of hilar obstruction, and hence bile obstruction is the implication of an anticipated cause of increased risk.^3^, ^18^ In addition, Bangarulingam *et al*.^12^ reported that 4% of PEC occurred despite the administration of AP to all patients. This is comparable with our results of 3.2% in the AP group and 3.9% in the non‐AP group. In contrast, Navaneethan *et al*.^9^ described comparably moderate rates of cholangitis (2.1%) when administering antibiotics to all patients undergoing ERCP for PSC, as did Ismail *et al*. (1.4%)^8^ and Alkhatib *et al*. (1%).^6^ None of these studies included a control group of PSC patients not receiving antibiotics. Previous studies on patients with PSC have shown other factors associated with an increased risk of PEC and adverse events. Factors such as symptomatic patients (obstructive jaundice), ERCP duration, acute procedure, and a vast number of interventions needed (e.g. dilation or stent placement) were reported to increase the risk of PEC.^6^, ^7^, ^12^, ^14^ These findings were partially confirmed by our results, indicating a higher risk of adverse events with long ERCP duration. This could potentially account for a surrogate measure of the interventions required during ERCP. However, we were unable to properly adjust our data for all risk factors for PEC, such as the number of interventions needed, which is a limitation. Previously unreported, we observed a higher risk of postprocedural infectious adverse events in older patients.

Several previous studies on standard ERCP procedures have explained the inability of AP to reduce PEC or postprocedural infectious adverse events, similar to our results. Both Ishigaki *et al*. and von Seth *et al*. reported no effect of AP on PEC.^10^, ^13^ A meta‐analysis by Bai *et al*. concluded that AP had no effect in reducing PEC in unselected patients, but it was unknown for higher risk patients such as those with PSC.^23^ Combined with the results of our study, the current use of general AP could be ineffective in preventing PEC. Again, this does not necessarily mean that patients with PSC do not require AP. Although as an unadjusted secondary outcome, we also found that the use of antibiotics as treatment during the first 30 days was more common in the group that did not receive AP. Unfortunately, the registry does not allow us to explain the indication for this antibiotic use. Theoretically, this could reduce the incidence of PEC if treatment was started with loose clinical signs and not registered as an infectious complication. This would, in turn, point to the conclusion that patients with PSC still need AP. Other theories have emerged that focus not on the presence or absence of AP but rather on the timing. Bai *et al*. discussed the timing of administration of antibiotics, which could be at the end or after the ERCP procedure, or perhaps even prolonged prophylaxis. To our knowledge, some clinicians do use prolonged prophylaxis as an alternate approach but its effectiveness remains unclear.

The strengths of this study are the validity of the GallRiks registry^21^ and the large cohort, representing a coverage of more than 90% of all ERCP procedures in Sweden,^20^ which to our knowledge currently is the largest cohort of ERCP procedures for patients with PSC. Data from this cohort are also more heterogeneous and clinically applicable, as they do not originate exclusively from specialized tertiary referral centers. As to be expected from retrospective and observational studies, our study contains several limitations. The use of AP has not been randomized, and there is a high risk of selection bias. During more complex procedures, the investigator is more likely to administer prophylaxis. It is not possible to determine from the data whether prophylaxis was administered for a certain reason or as part of a general policy at the endoscopic center. We were also unable to adjust for all known risk factors for PEC, such as dominant stricture, disease severity, and interventions such as stenting or dilatations. A final drawback of our study is that our interpretation could potentially comprise a Type 2 error.

## Conclusions

We could not detect any reduction in infectious or overall adverse events if prophylactic antibiotics were administered for ERCP procedures performed with the indication PSC. However, considering the limitations of this study and the greater risk of PEC, prophylaxis is probably still necessary, but perhaps with a different strategy.
